# Coastal radar as a tool for continuous and fine-scale monitoring of vessel activities of interest in the vicinity of marine protected areas

**DOI:** 10.1371/journal.pone.0269490

**Published:** 2022-07-15

**Authors:** Samantha Cope, Brendan Tougher, Jessica Morten, Cory Pukini, Virgil Zetterlind

**Affiliations:** 1 ProtectedSeas, Anthropocene Institute, Palo Alto, California, United States of America; 2 Channel Islands National Marine Sanctuary, Santa Barbara, California, United States of America; 3 California Marine Sanctuary Foundation, Monterey, California, United States of America; 4 Waitt Institute, La Jolla, California, United States of America; MARE – Marine and Environmental Sciences Centre, PORTUGAL

## Abstract

Marine protected areas (MPAs) are widely utilized for conservation of the world’s marine resources. Yet, compliance with MPA regulations remains difficult to measure because of limits to human resources and a lack of affordable technologies to automate monitoring over time. The Marine Monitor, an autonomous vessel monitoring, recording, and reporting system leveraging commercial off-the-shelf X-band marine radar to detect and track vessels, was used to monitor five nearshore California MPAs simultaneously and continuously to identify and compare site-specific use patterns over one year. Vessel tracks were classified into two movement patterns to capture likely fishing activity, “focal” or “linear”, that corresponded with local targeted species. Some illegal fishing potentially occurred at all sites (7–17% of tracks depending on site) most frequently on weekends and at mid-day, but the majority of activity occurred just outside the MPAs and in the near vicinity suggesting both a high level of compliance with regulations and awareness of MPA boundaries. Time spent engaged in potential fishing activity compared to track counts suggests that unique vessels may spend more time fishing inside area boundaries at some sites than others. The spatial distribution of activity shows distinct concentrations near MPA boundaries at all sites which strongly suggests vessels purposefully target the narrow area at the MPA boundary or “fish the line”, a potential acknowledgement of successful spillover. This activity increased significantly during some local fishing seasons. Concentration of activity at MPA boundaries highlights the importance of continuous monitoring at a high spatial and temporal resolution. Reporting of vessel behavior at a fine-scale using radar can help resource managers target enforcement efforts and understand human use patterns near coastal MPAs.

## Introduction

Marine protected areas (MPAs) have received increasing attention as effective management tools for conserving both marine ecosystems and the associated services they provide to society [1, 2]. Since MPA efficacy requires adequate enforcement of established regulations, there is a need for technologies to assist resource-constrained human patrol efforts and help measure MPA compliance [3]. A compounding difficulty in evaluating compliance can be a limited availability of data on small-scale fisheries activity which inhibits evaluation of realized fishing effort in nearshore areas [4].

One such area where the distribution of fishing effort is of concern is within the network of state-managed MPAs in California, USA [5]. California’s Marine Life Protection Act (MLPA) requires the state to evaluate and modify its collection of MPAs to increase the effectiveness of conservation measures (California Fish and Game Code § 2853). At the time of writing, the MPA network covers 16% of state waters [6], and the area of no-take MPAs, where take of marine resources and consumptive use are prohibited, has grown from roughly 0.25% to 9.4% since MLPA implementation [7]. The expansion of area covered by no-take MPAs has not negatively impacted fishery landings across the state overall [3], but the spatial distribution of fishing effort likely changed in response [8–10] which could have impacts at a smaller scale.

The phenomenon of "spillover", the export of biomass from within MPA boundaries, is one potential benefit of no-take MPAs to fisheries and can lead to a spatial concentration of fishing effort at MPA edges or "fishing the line" [11,12]. While this behavior can be interpreted as confirmation of successful spillover [13], it is also important to consider the potential cascading effects on non-target species through bycatch [10] and competitive displacement [14], some of which may only be detected on longer timescales [15]. With biomass of target species generally increasing inside MPA boundaries in California [3], this type of legal fishing effort has been observed at some California MPAs [16–19].

Illegal fishing, one facet of illegal, unreported and unregulated (IUU) fishing, occurs when resources are harvested where regulations have defined take as unlawful [20] and has been detected within some California MPAs [3, 21, 22]. The MLPA requires adequate enforcement to ensure that illegal fishing activity would not prevent the conservation benefits of designated MPAs from becoming realized [23]. Static MPA boundaries were chosen in the siting process in California to facilitate public awareness and compliance evaluation [24]. Since implementation of the MLPA, there has been a decrease in vessel presence within MPAs in some regions, but the magnitude of change varies by vessel type, suggesting a mixed response across different fisheries [25].

Previous studies in California and elsewhere have used different strategies to estimate fishing effort including interviews [5, 16, 26–29], logbooks [16, 17, 27, 30], shore- or aerial-based observation [16, 25, 31–33], and population modeling [21]. Participatory vessel tracking systems that require vessel cooperation, like the Automatic Identification System (AIS) and vessel monitoring systems (VMS), help document large-scale commercial fishing activity, but these technologies are not required or used by most state-managed small-scale fisheries in nearshore waters [34] including those in California [35]. Participatory methods of data collection can also introduce bias in reporting [12], and other methods like trap surveys [18, 19, 36] are not applicable to all gear types. Most of these monitoring strategies do not provide a continuous analysis of effort.

Developments in technologies for vessel monitoring are ongoing. Satellite-based solutions, including synthetic aperture radar (SAR), radio frequency (RF) detection, and optical imagery, used by Global Fishing Watch, HawkEye360, and others [37, 38], offer non-participatory methods but can be limited by temporal resolution, data processing requirements, and difficulty in detecting small vessels amongst waves and coastline features [39]. For small-scale activity in coastal areas, autonomous marine vehicles and drones have been used to monitor areas of interest [40, 41]. The need for technologies that can enhance management of small-scale fisheries worldwide is addressed by numerous international goals [42].

The objective of this research was to evaluate the spatial and temporal extent of potential fishing activity in the vicinity of California MPAs using shore-based marine X-band radar, a solution for tracking non-participatory vessels [43, 44]. Marine Monitor (M2) systems (https://protectedseas.net/marine-monitor-m2) strategically located onshore near no-take MPAs were used to track vessel activity autonomously and continuously via radar for one year. Addressing the following research questions provide an overview of vessel activity patterns near no-take MPAs: 1) are there discernable patterns of vessel activity within and near MPAs, including potential illegal fishing? 2) are there differences in vessel activity across different MPAs? and 3) at each MPA, are there differences in vessel activity across days of the week, time of the day, or fishing seasons? Activity was quantified within the MPA vicinity at each site which was further subdivided into three regions (inside the MPA, at the MPA boundary, outside the MPA) to evaluate if illegal fishing or fishing the line may be occurring and observe fine-scale differences in activity in relation to MPA boundaries.

## Methods

### Study sites

**Fig 1:**
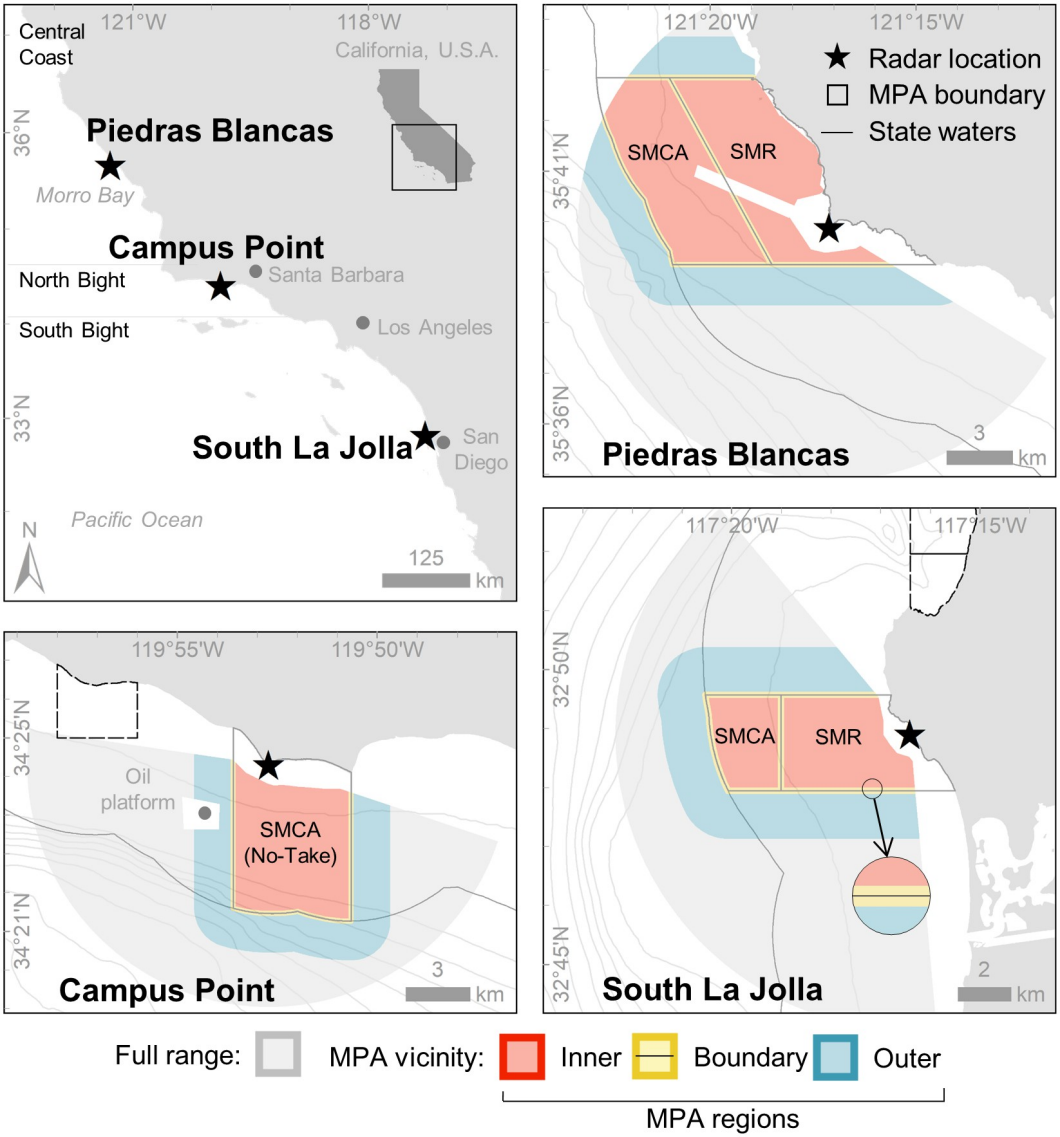
Study sites along the Central California Coast and within the Southern California Bight. MPAs include the Piedras Blancas State Marine Conservation Area (SMCA) and State Marine Reserve (SMR), Campus Point SMCA (No-Take), and South La Jolla SMCA and SMR. The full range of data collection at each site and MPA regions within each MPA vicinity are also shown. South La Jolla inset map shows detail of the MPA boundary region. Dashed lines show other MPAs not included in this research.

The MLPA requires a core set of no-take MPAs, but limited-take MPAs (often designated adjacent to a no-take area) have also been implemented in the network [24]. M2 systems monitored activity in the vicinity of both no-take and limited-take coastal MPAs in three distinct regions of the California coast—near Piedras Blancas, Campus Point, and South La Jolla (Fig 1). The no-take areas at each study site are classified as Tier I MPAs which are those identified by management to best provide evidence of the effects of protection over time [5]. No permits were required for work at these locations because data were collected with permission from private property.

The Piedras Blancas site is the most geographically remote of the study sites with the nearest marine enforcement based roughly 50 km away. The Piedras Blancas State Marine Reserve (SMR) and State Marine Conservation Area (SMCA) were established in 2007 to preserve an area of diverse habitats and species along the central coast [45]. While the take of all marine resources is prohibited in the SMR, the SMCA helps minimize the impact on local fisheries by allowing the commercial and recreational take of salmon and albacore.

The Campus Point site is less remote than Piedras Blancas with the city of Santa Barbara roughly 15 km away. Campus Point SMCA, created in 2012, is a no-take MPA with a single exception of take pursuant to maintaining artificial structures [45]. An offshore oil platform, installed in 1966 and located roughly 1 km outside the western boundary of the MPA, promotes fish assemblages and likely serves as nursery grounds for juvenile rockfish and other species [46]. The MPA protects areas of rocky reef and kelp forest, a preferred habitat of spiny lobster [47].

The MPAs at the South La Jolla site, just north of the city of San Diego, are adjacent to an area of high population density, thus more easily accessible compared to remote areas, and have some of the highest counts of reported MPA-related violations in the region [22]. South La Jolla SMR and SMCA were established in 2012 to preserve a rocky reef ecosystem that supports high biodiversity [45]. Presence of kelp forest and marine predators help prevent trophic cascades that can lead to ecosystem collapse [48]. All take of marine resources is prohibited in the SMR while the SMCA allows the recreational take of pelagic fin-fish by hook-and-line. The two MPAs were designated adjacent to each other, similar to those the Piedras Blancas site.

For this study, the MPA vicinity is defined at each site as the area within a 1-km buffer around the MPA boundaries as this is where fishing pressure near an MPA is likely greatest [36, 49]. This area is subdivided into inner, boundary, and outer MPA regions. A 200-m buffer centered on the MPA boundary line defines the boundary region where fishing the line typically occurs [27]. The inner and outer regions are the remaining areas within and outside the MPA, respectively. Activities of interest occurring within the inner region indicate potential illegal fishing activity. Activity was also measured in a spatial grid. Cell size (246 m, ~0.06 km2) was determined using the positional error model defined in [44]. These areas were clipped to each site-specific range of data collection, defined in the subsequent section.

### Vessel data collection

The M2 system uses commercial-off-the-shelf marine radar to detect and track target positions over time creating trajectories, or "tracks" (see [44] for detailed description). These tracks represent the path a vessel traveled. Spatial data recorded by M2 includes detection points with the following attributes: geolocation, timestamp, observed speed over ground, heading, and unique track record number. Detection points were most often recorded at two-second intervals. Initial data preparation included removal of duplicate detection points and points with missing attributes. Points were spatially clipped to site-specific detection ranges within 5 nautical miles (9.3 km) of the radar system where target tracking of small vessels is most reliable (https://protectedseas.net/marine-monitor-m2) (Fig 1). Radar technology relies on emitted electromagnetic pulse reflections to identify solid objects, so areas near the shoreline, physical structures, or known coastline and topographical obstructions were removed from analysis due to high likelihood of false detections.

One M2 system per study site collected data from 1 January 2019 through 31 December 2019 with some temporal monitoring gaps due to system maintenance. To reduce temporal bias in analysis, tracks that began on maintenance days and the day immediately prior and following were excluded. Unique track records were then counted per day at each site. Additionally, to exclude data where weather or other environmental conditions likely degraded monitoring performance, tracks that began on days with log-transformed daily track counts greater or less than two standard deviations above or below the site-specific daily average were not considered. This process resulted in the removal of 35%, 9%, and 13% of total days in 2019 from analysis at Piedras Blancas, Campus Point, and South La Jolla, respectively. The count of days removed at each step of data preparation and a breakdown of remaining "analysis days" by month can be found in S1 Appendix.

Finally, likely false targets caused by sea clutter and weather events, a common issue with data collected via marine radar [50], were removed from consideration using machine learning, a tool that has been used to classify trajectory patterns of fishing behavior [51, 52]. Following the process described in [53], ground truthed M2 tracks were used to train and tune a model which classified all track records as true vessels or false targets. An accuracy assessment showed that 95% of sample records were classified correctly. Records identified as false targets via the machine learning model were removed from analysis. See S1 Appendix for details. While some true vessel records may have been removed at this step, and some vessels may have gone undetected in rough surface conditions [50], the remaining data were sufficient to capture broad patterns of activity.

### Vessel activity and activities of interest

Vessel activity was quantified in three ways: counting unique tracks, summing cumulative track hours, and estimating the daily activity normalized by area for comparing across sites. Heuristic rules selected from published literature identified potential fishing activity that occurred during a track. Unlike participatory vessel tracking systems (e.g., AIS and VMS), radar technology does not provide the absolute identity of a target or direct information on activity. There is a growing body of research using speed and heading trajectory features of known fishing activity to identify specific trajectory patterns for estimating fishing effort [51, 54] or detecting abnormal fishing behaviors [55] via AIS or VMS trajectories. For our radar-based analysis, heuristic rules identify two "activities of interest" that reflected potential fishing behaviors—focal and linear—and were applied to tracks collected by M2. Rules were evaluated at the detection-level using instantaneous speed and heading. Additionally, calculating average speed and heading change across the preceding 12 detection points captured the temporal trend in activity at each point [56, 57].

**Fig 2:**
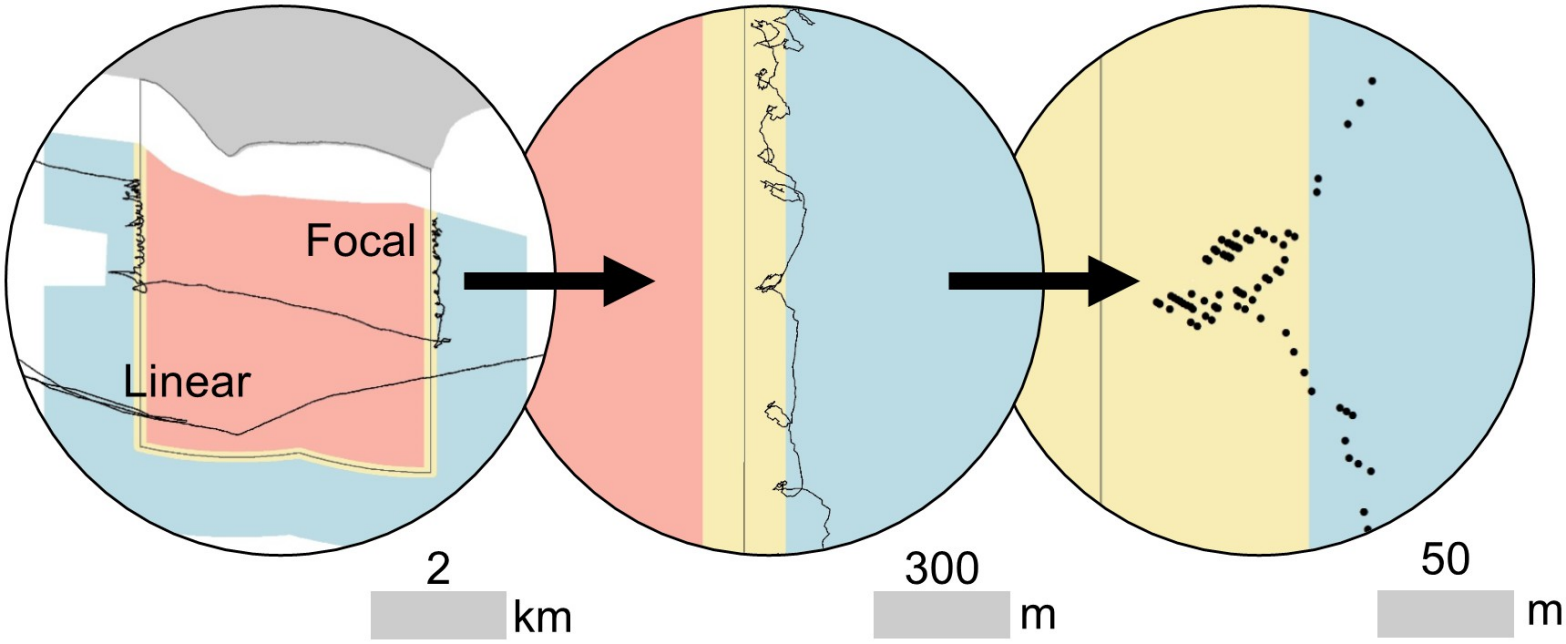
Examples of focal and linear trajectory patterns. Red, yellow, and blue areas indicate the inner, boundary, and outer MPA regions, respectively. From left to right: full track records within the MPA regions, magnified view of the focal activity track, and detection points in the boundary region are shown with the respective scale.

- A **focalized trajectory** (hereafter referred to as focal activity) represents pauses in motion, idling, or fine-scale maneuvering. This pattern was identified by low speed and/or moderate to high directional changes as follows: 1) instantaneous speed was between 2 and 6 knots (1 and 3 m/s), and 2) instantaneous heading changed by greater than or equal to 15-degrees from the preceding average, or 3) instantaneous speed was less than 2 knots (1 m/s). Based on previous research, these rules identify targets potentially engaged in trapping [58, 59], hauling with nets or fishing with hook-and-line [54, 60], or non-transitory recreational activity [61].
- A **linear trajectory** (hereafter referred to as linear activity) is characterized by slow constant speed in a consistent direction. This pattern was identified as follows: 1) instantaneous speed was between 2 and 6 knots (1 and 3 m/s), and 2) instantaneous heading changed by less than 15-degrees from the preceding average, and 3) instantaneous speed was under 25% more or less than the preceding average. These rules aim to identify targets engaged in potential trawling [51, 62] or trolling [63]. This definition assumes that transit activity occurs at speeds greater than 6 knots (3 m/s), but it is possible that some slower transitory or survey-type activities were captured by this pattern. See Fig 2 for pattern examples.

Only points defined as focal or linear within a consecutive grouping of 12 or more detections were classified as track segments over which an activity of interest occurred under the assumption that 12 detections were necessary to describe an activity [56]. Points that were not classified as focal or linear activities (likely non-fishing) were retained for calculating overall vessel activity but were not included in potential fishing analysis. All analyses were conducted in R [64].

#### Track counts

Unique track records were grouped and summed by site, spatial region, and classified activity of interest for each analysis day. It is important to note that a single unique vessel may have been represented by multiple tracks if there was a disruption in radar tracking due to rough surface conditions or if the vessel exited and re-entered the range of radar detection. Thus, track counts may overestimate unique vessels that were present.

#### Track hours

Calculating the duration of tracks provides an estimate of activity independent of the number of vessels present. Consecutive detection points along a unique track were clipped to the relevant spatial unit (MPA vicinity, MPA region, spatial grid), and duration calculated using the difference in time between the first and last point [65]. Values were summed across tracks regardless of temporal overlap ("track hours") to best reflect the cumulative pressure on an area, similar to [66], as it takes multiple concurrent tracks into account. Track hours were summed across each analysis day for each site, spatial region, and activity of interest.

#### Daily activity normalized by area

Daily track hours were normalized by dividing by the area of the relevant spatial unit [66]. This activity metric (daily hours per km^2^) facilitates comparisons across sites and MPA regions taking into account differences in area observed.

### Temporal analysis

To broadly assess the number of vessels accessing MPA regions over time, tracks over which an activity of interest occurred were assigned two temporal attributes using the timestamp of the first detection point: day of the week and hour of the day. Unique tracks were summed across each analysis day at both temporal scales.

For a statistical analysis of daily activity of interest compared to fishing seasons, detection points classified as focal or linear were assigned to closed/open fishing seasons using their timestamp. Common commercial and recreational fisheries with seasonal closures operating in the respective regions [67] were associated with focal or linear patterns (Table 1) based on allowed gears per fishery (S1 Table). Only those fisheries with allowed gear types matching the activity pattern of interest were considered (e.g., trapping classified as focal, trawling classified as linear). Fisheries without seasonal closures were not included in analysis but may have conflated results, as later discussed. Differences across closed/open fishing seasons using exclusively daytime, night time, weekday, or weekend activity were also evaluated. Points were classified day/night using the StreamMetabolism package [68] based on site-specific sunrise and sunset times. Points occurring Monday-Friday were classified as weekday; those occurring Saturday-Sunday were classified as weekend.

**Table 1:** Common commercial and recreational fisheries with seasonal closures at applicable sites.

	Type	Piedras Blancas		Coal Oil Point		South La Jolla	
		Focal	Linear	Focal	Linear	Focal	Linear
**Dungeness crab**	Commercial	X					
	Recreational	X					
**Market squid**	Commercial	X		X		X	
**Spiny lobster**	Commercial			X		X	
	Recreational			X		X	
**Spot prawn**	Commercial	X		X		X	
**Nearshore fishery**	Commercial	X	X	X	X	X	X
**Groundfish**	Recreational	X	X	X	X	X	X
**Salmon**	Commercial	X	X				
	Recreational	X	X				
**White seabass**	Commercial	X	X				
**Pink shrimp**	Commercial		X				
**Ridgeback prawn**	Commercial		X		X		X
**California halibut**	Commercial				X		

More information on specific species, active dates (season was open), allowed gears, and regulation citations per fishery included in S1 Table.

Daily activity of interest within most groups was not normally distributed due to a high presence of days with minimal activity, potentially a result of weather events, seasonality, or other factors, as later discussed. These data could not be successfully transformed to fit a normal distribution even after removing days without activity of interest from respective analysis. As a result, differences in the magnitude of median daily activity across closed/open fishing seasons within each region were tested, ignoring days when no activity occurred, using the non-parametric exact two-sample quantile test (*q* = 0.5) [69, 70] via the snpar package [71]. See S2 Appendix for site-specific day counts when activities of interest occurred.

## Results

### Study sites

After removing days of incomplete data collection, 236, 332, and 319 analysis days were retained at the Piedras Blancas, Campus Point, and South La Jolla sites, respectively. Overall, there was roughly 388 km^2^ of marine area monitored and 74 km^2^ monitored within MPAs (Table 2).

**Table 2:** Marine area (km^2^) monitored at each site.

	Piedras Blancas	Campus Point	South La Jolla	Overall
**Full range**	156.74	123.95	107.60	388.29
**MPA vicinity**	69.96	47.81	41.42	159.19
**Inner MPA region**	38.06	21.23	15.18	74.47

### Track counts

Between 60–80% of all tracks observed (depending on site) entered the MPA vicinity while only 15–30% of all tracks exhibited an activity of interest (potential fishing) (Table 3). In general, the number of potential fishing tracks per day was greatest at South La Jolla and lowest at Piedras Blancas. Overall, 10% of all tracks observed exhibited an activity of interest within the inner MPA compared to 23% in the greater MPA vicinity suggesting that the majority of fishing activity near the MPA was likely legal activity.

**Table 3:** Track counts across analysis days at each site and overall.

	Piedras Blancas		Campus Point		South La Jolla		Overall
	Sum	Daily	Sum	Daily	Sum	Daily	Sum
**All observed**	2,407	10±10	14,136	43±15	15,347	48±27	31,890
Likely non-fishing	1,439	6±6	11,176	34±12	7,353	23±13	19,968
Potential fishing	968 (40%)	4±4	2,960 (21%)	9±6	7,994 (52%)	25±15	11,922 (37%)
Focal	502	2±2	2,252	7±5	5,697	18±12	8,451
Linear	874	4±4	2,085	6±4	6,570	21±13	9,529
**Outside MPA vicinity**	1,595 (66%)	7±6	11,169 (79%)	34±11	10,545 (69%)	33±21	23,309 (73%)
Likely non-fishing	1,055	4±5	9,551	29±10	5,767	18±12	16,373
Potential fishing	540 (22%)	2±3	1,618 (11%)	5±4	4,778 (31%)	15±11	6,936 (22%)
Focal	268	1±2	1,125	3±3	3,082	10±7	4,475
Linear	502	2±3	1,235	4±3	4,066	13±10	5,803
**MPA vicinity**	1,916 (80%)	8±8	8,424 (60%)	25±13	10,540 (69%)	33±19	20,880 (65%)
Likely non-fishing	1,268	5±6	6,299	19±10	5,974	19±11	13,541
Potential fishing	648 (27%)	4±3	2,125 (15%)	7±4	4,566 (30%)	15±9	7,329 (23%)
Focal	296	1±2	1,565	5±4	3,289	10±7	5,150
Linear	573	2±3	1,443	4±3	3,556	11±7	5,572
**Inner MPA region**	1,577 (66%)	7±7	6,245 (44%)	19±11	6,832 (45%)	21±15	14,654 (46%)
Likely non-fishing	1,166	5±5	5,286	16±10	4,952	16±11	11,404
Potential fishing^a^	411 (17%)	2±2	959 (7%)	3±3	1,880 (12%)	6±5	3,250 (10%)
Focal	149	1±1	549	2±2	1,189	4±3	1,887
Linear	349	1±2	697	2±2	1,418	4±4	2,464

Total track count (Sum) and average daily track count with standard deviation (Daily). Tracks exhibiting both focal and linear activity are counted in both categories. Parentheses indicate the percentage of all tracks observed across the full range (first row). Values have been rounded to the nearest whole number.

^a^ Potential fishing within the inner MPA region represents potential illegal fishing.

### Track hours

Roughly half of all track hours occurred within the MPA vicinity at all sites while only 18–22% of all track hours were spent potentially fishing (Table 4). Similar to track counts, Piedras Blancas had the lowest hours per day observed in general. Track hours per day were roughly similar at Campus Point and South La Jolla in the MPA vicinity and within the inner MPA. Overall, only 6% of all track hours were spent potentially fishing within the inner MPA region.

**Table 4:** Total track hours on analysis days at each site.

	Piedras Blancas		Campus Point		South La Jolla		Overall
	Sum	Daily	Sum	Daily	Sum	Daily	Sum
**All observed**	1,346	6±6	4,259	13±6	4,220	13±8	9,825
Likely non-fishing	728	3±3	2,681	8±4	2,271	7±4	5,680
Potential fishing	618 (46%)	3±3	1,578 (37%)	5±4	1,949 (46%)	6±4	4,145 (42%)
Focal	321	1±2	1,048	3±3	1,262	4±3	2,631
Linear	298	1±2	530	2±2	687	2±2	1,515
**Outside MPA vicinity**	728 (54%)	3±3	2,232 (52%)	7±4	2,172 (51%)	7±5	5,132 (52%)
Likely non-fishing	356	2±2	1,458	4±2	1,156	4±2	2,970
Potential fishing	373 (28%)	2±2	773 (18%)	2±2	1,015 (24%)	3±3	2,161 (22%)
Focal	194	1±2	481	1±2	621	2±2	1,296
Linear	178	1±1	293	1±1	394	1±1	865
**MPA vicinity**	617 (46%)	3±3	2,020 (47%)	6±4	2,042 (48%)	6±4	4,679 (48%)
Likely non-fishing	373	2±2	1,223	4±2	1,119	4±2	2,715
Potential fishing	244 (18%)	1±2	797 (19%)	3±2	923 (22%)	3±2	1,964 (20%)
Focal	126	1±1	567	2±2	639	2±2	1,332
Linear	118	< 1	230	1±1	285	1±1	633
**Inner MPA region**	300 (22%)	1±1	822 (19%)	2±2	743 (18%)	2±2	1,865 (19%)
Likely non-fishing	219	1±1	639	2±1	456	1±1	1,314
Potential fishing^a^	81 (6%)	<1	183 (4%)	1±1	287 (7%)	1±1	551 s(6%)
Focal	24	<1	93	<1	195	1±1	312
Linear	57	<1	90	<1	91	<1	238

Total track hours (Sum) and average daily track hours with standard deviation (Daily). Parentheses indicate the percentage of all track hours observed across the full range (first row). Values have been rounded to the nearest whole number. Note that the cumulative summation reflects total track hours regardless of temporal overlap.

^a^ Potential fishing within the inner MPA region represents potential illegal fishing.

**Fig 3:**
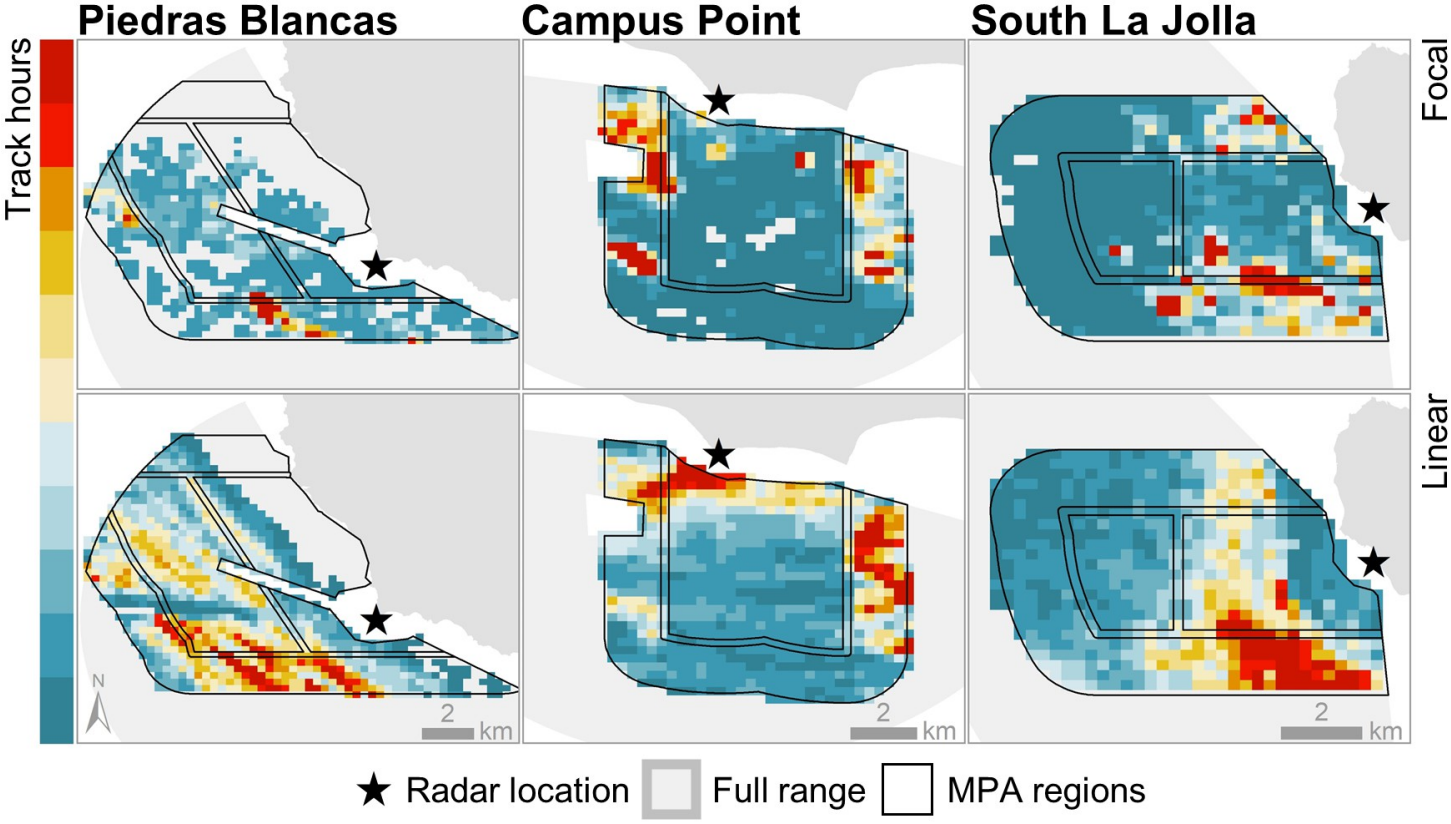
Total track hours across spatial grid. Black lines show extent of MPA regions. Blue to red shading indicates low to high values, respectively. Values have been normalized for each site and activity of interest to highlight spatial patterns.

Distinct concentrations of activities of interest (hot spots) were visible at all sites (Fig 3) with activity occurring primarily on one side of the MPAs at Piedras Blancas and South La Jolla and on two sides of the MPA at Campus Point. Areas where focal activity was concentrated generally occurred near the MPA boundary line, although some areas were also within the inner MPA region at Campus Point and South La Jolla.

### Daily activity normalized by area

**Fig 4:**
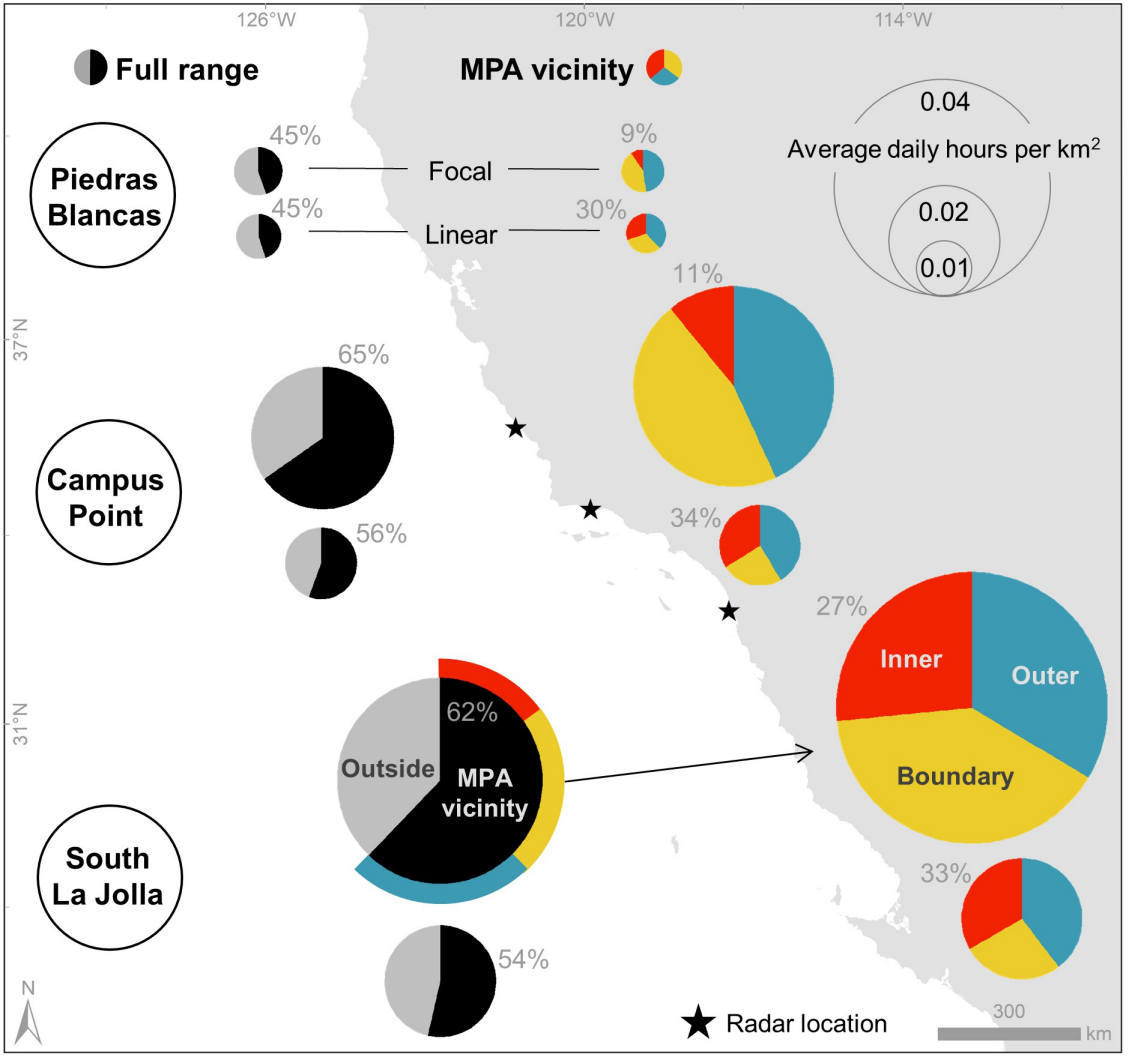
Daily activity of interest normalized by area. Pie chart size indicates average daily hours per km^2^ of either focal or linear activity across the full range (left, further classified by activity occurring within and outside MPA vicinity) and MPA vicinity (right, further classified by activity occurring with MPA regions). Percent within the MPA vicinity is noted in full range pie charts; percent within the inner MPA region is noted in MPA vicinity pie chart. See S2 Appendix for all values.

At Campus Point and South La Jolla, a majority of activity occurred within the MPA vicinity; there was a lesser share at Piedras Blancas (Fig 4). Average daily focal and linear activity was greatest at South La Jolla and lowest at Piedras Blancas which was to be expected given the large difference in remoteness between the sites. Daily focal activity occurred most often within the boundary and outer regions at all sites, but less so at South La Jolla where activity within the inner region made up a larger percent than at other sites. At South La Jolla, 27% of focal activity in the MPA vicinity occurred within the inner region while only 9% and 11% occurred within the inner MPA at Piedras Blancas and Campus Point, respectively. Linear activity was more consistent across all MPA regions at all sites.

### Temporal analysis

**Fig 5:**
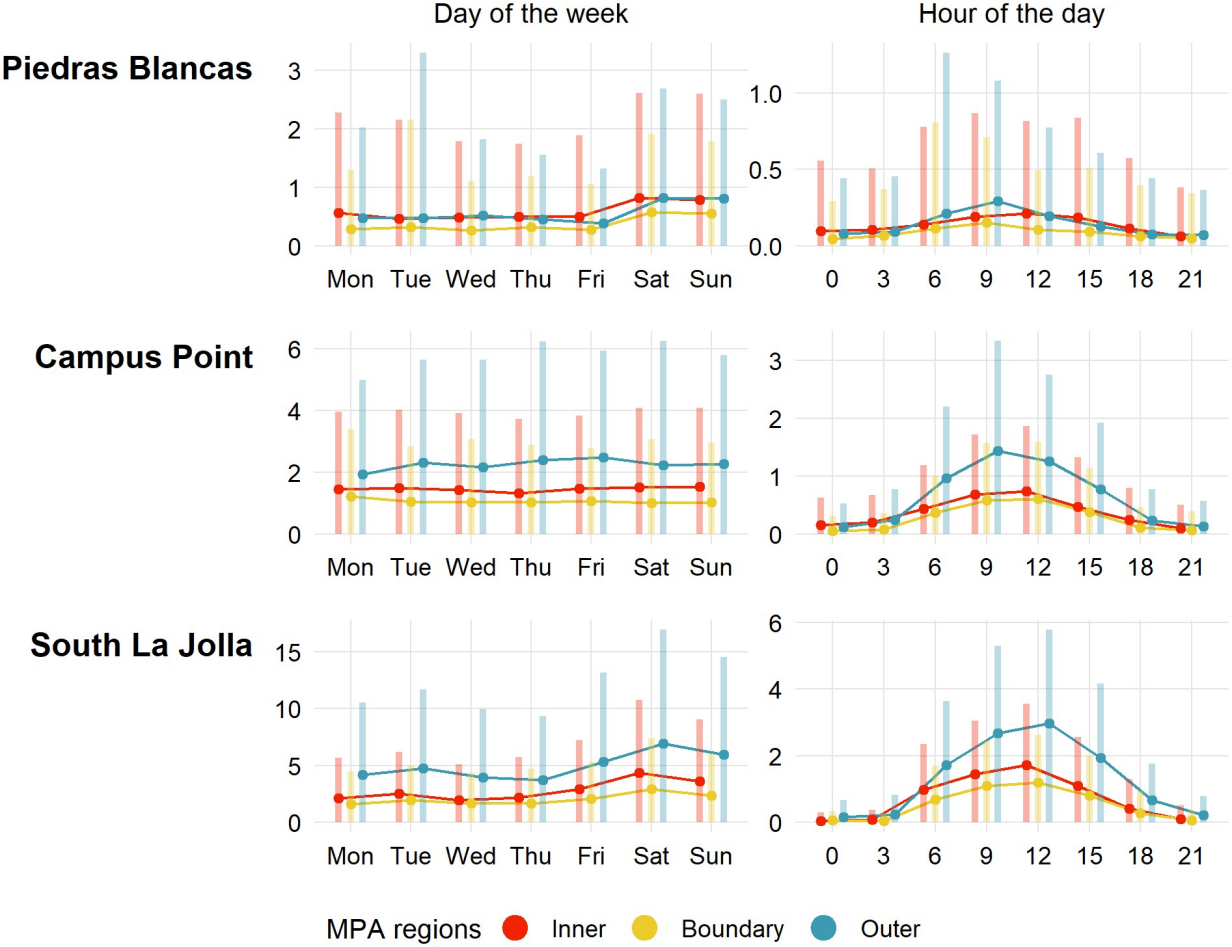
Daily tracks over which an activity of interest occurred by day and time. Points indicate daily average; bars indicate standard deviation (portions below zero are not shown).

The number of tracks per day over which potential illegal fishing occurred peaked on weekends at Piedras Blancas and South La Jolla with an average of roughly 1 and 5 tracks per day on Saturday at Piedras Blancas and South La Jolla, respectively (Fig 5). The number of tracks per day was more consistent across days of the week at Campus Point. Tracks occurred most often during daylight hours at all sites with potential illegal fishing activity peaking between 12:00 and 15:00.

**Fig 6:**
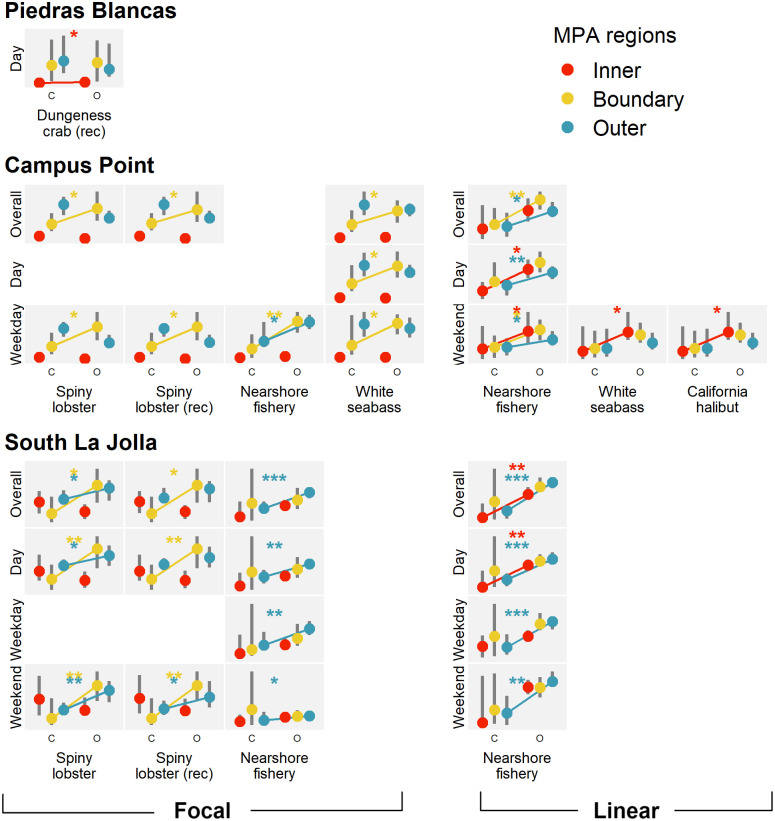
Differences in activity between closed and open fishing seasons. Small grey boxes depict differences between closed (left side (C)) and open (right side (O)) seasons by fishery (x-axis) and time (y-axis) at each site. Points indicate the median daily hours per km^2^ in MPA regions with the vertical grey bars showing the spread of median values within the 95% confidence interval. Values have been normalized to facilitate comparison across fisheries. Lines indicate significant differences across groups (*p* < 0.05*, *p* < 0.01**, *p* < 0.001***) when the median in the open season was greater than that in the closed. Only those fisheries with at least one significant difference are shown. Sample sizes for these comparisons ranged from *n* = 22 to *n* = 44 at Piedras Blancas, *n* = 14 to *n* = 250 at Campus Point, and *n* = 6 to *n* = 266 at South La Jolla.) See S3 Appendix for the specific sample sizes of all unique groups.

Fisheries with significantly greater daily activity of interest during the open season within at least one MPA region are shown in Fig 6; activity in other respective regions are shown for context. There were few significant differences at Piedras Blancas. Low sample sizes at this site (see S3 Appendix), a result of low data reception for much of January through March, prevented comparisons across closed/open commercial Dungeness crab, recreational groundfish, and market squid seasons primarily at night and on weekends.

Daily focal activity at Campus Point was significantly greater during multiple open seasons primarily within the boundary region on weekdays while activity in the other regions was typically less. At South La Jolla, focal activity was significantly greater during open spiny lobster seasons primarily in the boundary and outer regions during the day and on weekends. In addition, there was significantly greater focal and linear activity during the open commercial nearshore fishery season primarily in the outer region, but linear activity was also significantly greater during the open season within the inner region.

## Discussion

This research demonstrates the ability to capture and classify vessel behavior using radar and establishes upper bounds on potential fishing activity in and around MPAs. Broad patterns were common across all monitored sites, the most striking commonality being that potential fishing occurred primarily near and at the boundary of MPAs relative to inside the MPA which suggests a spatial awareness of the boundary line and by implication awareness of MPA restrictions. Given that regulations for the included MPAs exclusively limit extractive activities (not transit or other non-consumptive uses), vessels that are not engaged in fishing have no clear incentive to avoid the MPA. Thus, it is likely that focal or linear definitions captured fishing activity.

Focal activity hot spots were also evident in the inner MPA region. Results at Piedras Blancas suggest that vessels engaged in potential fishing activity could be "cutting the corner" of the SMCA where high concentrations of linear activity occurred, although trolling for salmon and albacore is allowed in this area [45]. While fishing at night is one method used for evading detection [72], results presented here indicate that night time activity was generally low at these sites with potential fishing peaking at mid-day.

Over half of all tracks observed (65%) passed through the MPA vicinity. Average daily track counts were roughly similar at Campus Point and South La Jolla (43 and 48, respectively), but there were roughly twice as many daily potential fishing tracks on average observed at South La Jolla within the full range, MPA vicinity, and inner MPA region. Given the proximity of the South La Jolla site to San Diego, the higher number of unique vessels engaged in potential fishing activity was expected. At a greater distance from population centers, Piedras Blancas had average daily track counts that were consistently lower than the other sites.

Fewer than half of all track hours (48%) occurred in the MPA vicinity. While there were generally more potential fishing tracks observed at South La Jolla than the other sites, average daily track hours were roughly similar at Campus Point and South La Jolla. Potential illegal fishing occurred on average for 1 hour per day at both sites and over 3 and 6 unique tracks at Campus Point and South La Jolla, respectively. This suggests that individual vessels engaged in potential fishing activity for a longer duration at Campus Point compared to individual vessels at South La Jolla, and both scenarios have different implications for management and local ecosystems. For example, vessels fishing illegally at South La Jolla may be easier to apprehend given the greater number, but disrupting an incident of illegal fishing at Campus Point may reduce pressure on the ecosystem to a greater extent given the longer duration of each event.

Thus, it is important to acknowledge the difference between the count of unique tracks and the magnitude of track hours. While 10% of all tracks observed exhibited potential fishing activity inside the inner region, only 6% of all track hours occurred in the inner MPA region suggesting regular compliance with regulations at these MPAs. The difference between track counts and hours suggests that vessels may briefly enter the inner MPA region but spend the majority of time at the boundary and just outside the MPA.

The spatial distribution and amount of activity at Campus Point and South La Jolla reflect more broad access from major ports and metropolitan areas than Piedras Blancas. Despite fewer tracks and track hours observed per day at the Piedras Blancas site compared to other sites, focal activity was still concentrated at the boundary. Previous research along the central coast indicates that fishing did not occur in the vicinity of a remote MPA, but the authors hypothesized that increased species biomass within the MPA could have resulted in spillover and thus offsetting the cost of greater travel times from port [31]. Results showed that daily activity normalized by area was more common outside the MPAs at Piedras Blancas, but vessels did access the MPA regions primarily at the southern boundary, likely traveling from Morro Bay, roughly 50 km away.

Concentrated activity at MPA boundaries is potentially an acknowledgement and confirmation of successful spillover from within MPAs [13]. Analysis of daily hours normalized by area showed that vessels engaged in potential fishing activity spend more time within the MPA vicinity than outside at Campus Point and South La Jolla, and the spatial arrangement of activity hot spots just outside MPA boundaries at all sites, including Piedras Blancas, suggests fishers target spillover. The concentration of vessel activity within the MPA vicinity could indicate that the benefits of MPA designation at these California study sites are becoming realized, although there may be a number of factors that influence vessel activity near MPAs [33].

**Fig 7:**
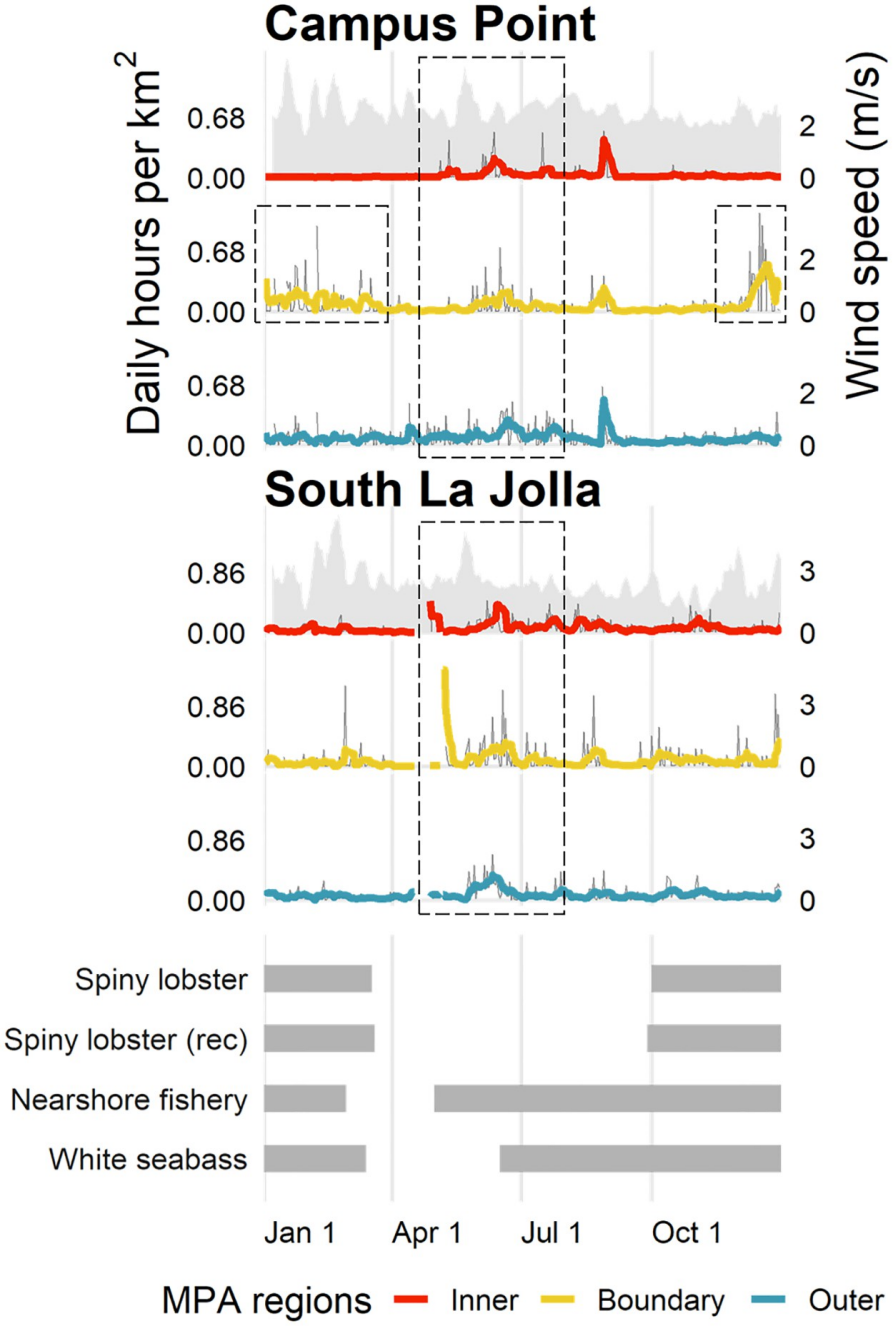
Activity over time. Daily hours per km^2^ of focal activity in MPA regions at Campus Point and South La Jolla. Red, yellow, and blue lines show a 7-day running average within the inner, boundary, and outer MPA regions, respectively. Gaps resulted from days without data collection removed from analysis. Shaded region shows a 7-day running average of wind speed for reference (collected for accuracy assessment; see S1 Appendix). Dashed lines highlight activity of interest related to open fishing seasons. Bar graphs (bottom) show the temporal extent of open fishing seasons of interest at these sites (see S1 Fig for a similar figure including linear activity and all sites and fisheries).

Temporal analysis of daily activity across closed and open fishing seasons strongly suggests that fishing the line occurred. This behavior was most pervasive at Campus Point as the focal activity within the boundary region was significantly greater during the open spiny lobster and white seabass seasons, while activity within the other MPA regions was less (Fig 6). At South La Jolla, there was significantly greater focal activity primarily in both the boundary and outer regions when lobster and nearshore fishery seasons were open suggesting less attention to the boundary line specifically. Less activity occurring within the inner region during the lobster season suggests that lobster harvesting is likely not a main component of illegal activity at South La Jolla. The commercial and recreational lobster seasons are also open primarily during winter months when recreational fishing effort is less than during summer/fall [16]. These trends in focal activity are visible when looking at daily activity of interest over time compared to fishing seasons (Fig 7).

More unique vessels likely participate in illegal fishing activity at South La Jolla than at other sites. Most areas with a high concentration of potential fishing activity at South La Jolla were within the SMR (Fig 4) where all take is prohibited and MPA-related citations have been common [22]. Recreational vessels likely made up a large portion of the potential fishing activity observed. Some of the highest levels of recreational fishing effort statewide occur in the vicinity of the South La Jolla site [5] with recreational vessels more commonly fishing within MPA boundaries than commercial vessels [33]. Previous research indicates that recreational fishing more commonly occurs on weekends than weekdays in the area [16], a pattern that was also evident in potential fishing activity results presented here. It is important to note that small, low-profile craft, like kayaks, may not have been captured by radar but are active in southern California [73].

Concentration of activity near MPAs underscores the importance of compliance monitoring and enforcement when required. While the temporal resolution of some participatory tracking systems, like VMS, can be on the order of minutes or hours [54] preventing small-scale applications [74], the resolution of the radar data provided by the M2 system, on the order of seconds, facilitates analysis of activities occurring within small geographic areas. Previous research conducted at South La Jolla SMR utilizing trap surveys found that while the number of traps decreased after MPA designation, the distribution became more clustered at the boundaries [18]. Recent work in the vicinity of Campus Point SMCA used catch reporting at the scale of a fishing block designated by the managing authority (~140 km^2^) and determined catch is greater in the block containing the MPA [19]. Results from radar tracking, presented here, provide greater detail on the spatial distribution of effort within that unique fishing block.

With non-consumptive use within the vicinity of MPAs becoming increasingly common in the southern California region [33], the defined heuristic rules chosen could have captured vessels not engaged in fishing. The linear behavior, besides capturing trawling or trolling, is also consistent with slow transit, a common mode exhibited by smaller recreational vessels and sailboats to reduce fuel consumption or due to inherent limits in propulsion. Further analysis could link trajectory patterns detected by radar to specific fishing gear using photographs or onsite observers. Previous research was utilized that largely relied on AIS and VMS data, so performing similar efforts using radar tracking in the future could likely illuminate more distinctive fine-scale patterns associated with small-scale fishing effort.

Temporal analysis across fishing seasons is limited by the binary classification of closed/open seasons. Vessels targeting commonly fished species without seasonal closures, such as rock crab [67], were likely tracked by radar, but the relationships between season and activity could not be captured, and their presence could have conflated analysis. The broad classification scheme also did not account for variation within distinct fishing seasons (Fig 7) when the magnitude of activity could fluctuate based on other factors, such as holidays or weather events. Previous research found that illegal fishing activity is most likely to occur on days with traditionally good boating conditions, a combination of little rain, low wind speeds, and calm surface conditions [75]. The distinct opening and/or closing days of fishing seasons can also influence fishing effort [12, 18, 47]. The small sample size of activity days at Piedras Blancas overall and at all sites during some closed and open fishing seasons at night, likely due to low activity, inhibited robust analysis at some scales.

## Conclusions

As more becomes known about the spatial and temporal patterns of vessel activity near MPAs, it will be important to evaluate the biological impacts of illegal fishing (10% of all tracks observed in this research) and fishing the line (23% of all tracks) and adapt management design if necessary. There is some evidence that incentive-based individual fishing quotas, implemented in a commercial fishery along the U.S. west coast, motivate voluntary avoidance of unintended catch at area boundaries [76]. Increasing MPA size could expand the area of protected habitat with potential benefit to fisheries, although this is dependent on additional biological and economic variables [77]. Under the existing management regime at the time of writing, patterns of vessel activity revealed in this research suggest that enforcement efforts could intercept the greatest number of vessels potentially fishing illegally with patrols during daylight hours and near MPA boundary lines.

Implementation of the MLPA led to the formation of California’s interconnected MPA network distributed along the state’s expansive 840-mile (1,350-km) coastline. Generally regarded a success, the network created a foundation for marine conservation in state waters by increasing the area and habitats protected [3, 78]. But it is important that recognition be paired with effective monitoring and enforcement to ensure the intended benefits are realized [79]. Radar tracking via the M2 system provided high spatial and temporal resolution with minimal physical resources and human effort required—called for by [3] and others. Data collected this way can alert managers to activity within MPA boundaries and reveal other spatial factors that influence fishing effort. A clearer picture of fishing activity provides valuable detail when estimating effort and harvest, ultimately facilitating more informed evaluations of impact on both fisheries and species of interest within established MPAs.

## Supporting information

S1 Fig Activity of interest and fishing seasons over time. (PDF)

S1 Table Fisheries with seasonal closures definitions. (PDF)

S1 Appendix Data preparation and false target identification details. (PDF)

S2 Appendix Daily activity detailed results. (PDF)

S3 Appendix Temporal analysis sample sizes at all sites. (PDF)
