# Supplementary material for: Coastal radar as a tool for continuous and fine-scale monitoring of vessel activities of interest in the vicinity of marine protected areas
Source: PLoS One. 2022 Jul 15;17(7):e0269490. doi: 10.1371/journal.pone.0269490 (PMC9286260; doi:10.1371/journal.pone.0269490)
Supplement: S1 Fig — (PDF) [file pone.0269490.s001.pdf]

S1 Fig. Activity of interest and fishing seasons over time.

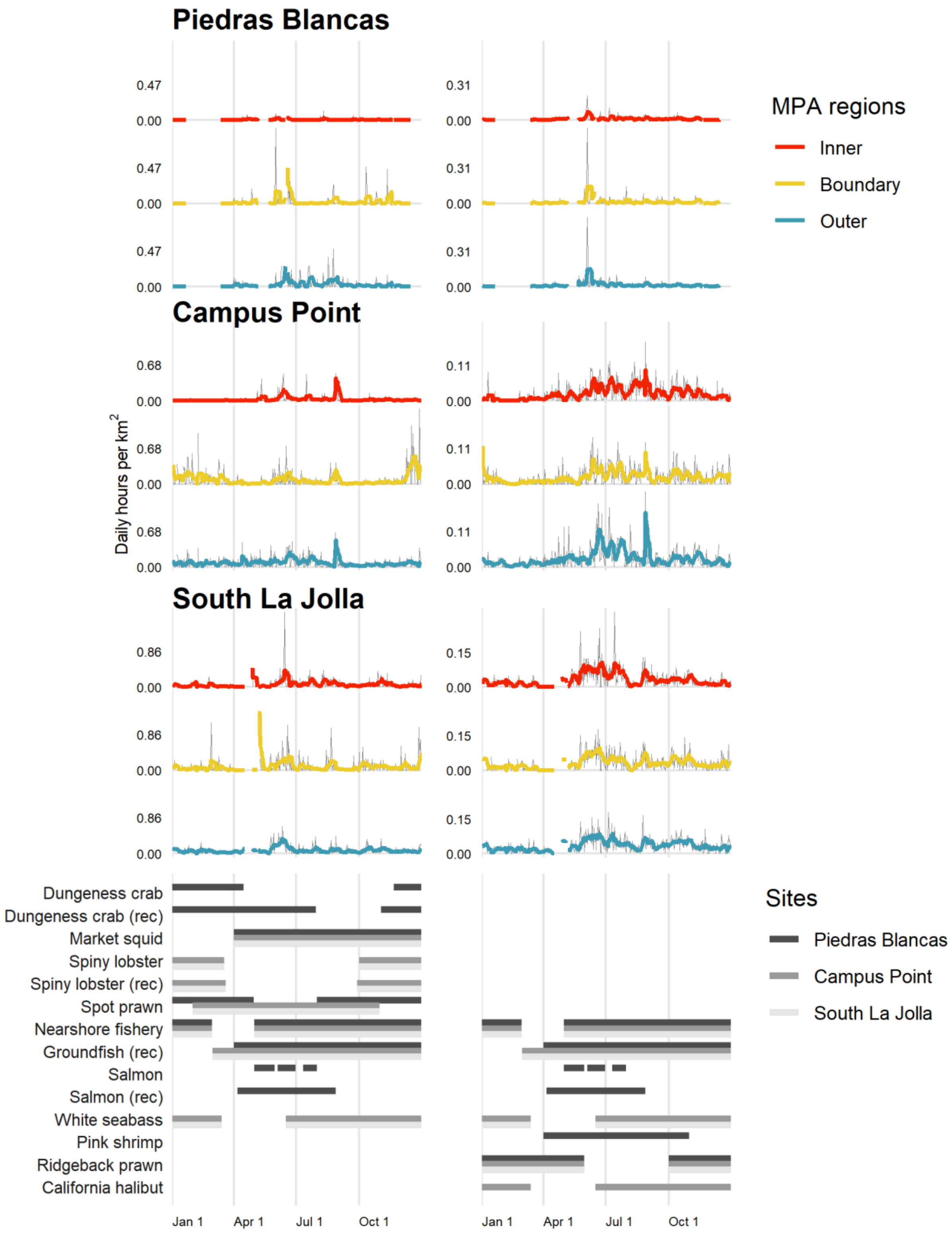

Daily hours per km^2 of focal (left) or linear (right) activity in MPA regions. Red, yellow, and blue lines show a 7-day running average within the inner, boundary, and outer MPA regions, respectively. Gaps resulted from days without data collection removed from analysis. Bar graphs (bottom) show the temporal extent of open fishing seasons in 2019 per site.
