# Supplementary material for: Coastal radar as a tool for continuous and fine-scale monitoring of vessel activities of interest in the vicinity of marine protected areas
Source: PLoS One. 2022 Jul 15;17(7):e0269490. doi: 10.1371/journal.pone.0269490 (PMC9286260; doi:10.1371/journal.pone.0269490)
Supplement: S1 Table — (PDF) [file pone.0269490.s002.pdf]

S1 Table. Fisheries with seasonal closures definitions.

Fishery	Type	Site	Active dates	Allowed gears
Spiny lobster (*Panulirus interruptus*)	Commercial	CP<br>SJ	1 January–18 March,<br>2 October–31 December[a]<br>(14 CCR § 121)	Lobster trap (Fish and Game Code Section 9010(a))
	Recreational		1 January–20 March,<br>28 September–31 December<br>(14 CCR § 29.90)	Hoop net, by hand<br>(14 CCR § 29.80)
Pink shrimp (*Pandalus jordani*)	Commercial	PB	1 March–31 October<br>(14 CCR § 120.1)	Trawl<br>(14 CCR § 120)
Dungeness crab (*Metacarcinus magister*)	Commercial	PB	1 January–15 April,<br>22 November–31 December[b]	Dungeness crab trap (Fish and Game Code Section 9011(a)(1))
	Recreational		1 January–30 July,<br>2 November–31 December<br>(14 CCR § 29.85)	Hoop net, by hand, trap<br>(14 CCR § 29.80)
Spot prawn (*Pandalus platyceros*)	Commercial	PB<br><br><br><br><br>CP<br>SJ	1 January–30 April,<br>1 August–31 December[c]<br>(14 CCR § 180.1)<br><br>1 February–31 October[d]<br>(14 CCR § 180.1)	Trap<br>(14 CCR § 180.1)
Nearshore fishery (defined by 14 CCR § 1.90)	Commercial	PB<br>CP<br>SJ	1 January–28 February,<br>1 May–31 December<br>(50 CFR Part 660)	Dip nets, hook-and-line, trap<br>(14 CCR § 150(l))

Groundfish (defined by 14 CCR § 1.91)	Recreational	PB<br><br><br><br>CP<br>SJ	1 April–31 December (14 CCR § 27.40)<br><br>1 March–31 December (14 CCR § 27.45)	Hook-and-line, by hand (14 CCR § 28.65)
Ridgeback prawn (*Sicyonia ingentis*)	Commercial	PB<br>CP<br>SJ	1 January–31 May, 1 October–31 December (14 CCR § 120.3)	Trawl (14 CCR § 120)
White seabass (*Atractoscion nobilis*)	Commercial	CP<br>SJ	1 January–14 March, 16 June–31 December (14 CCR § 155)	Gill/trammel net, hook-and-line (14 CCR § 155)
California halibut (*Paralichthys californicus*)	Commercial	CP	1 January–14 March, 16 June–31 December (Fish and Game Code Section 8496(a))	Trawl (Fish and Game Code Section 8495(a))
Salmon (*Oncorhynchus spp.*)	Commercial	PB	1 May–31 May, 4 June–30 June, 11 July–31 July[e]	Hook-and-line (14 CCR § 182)
	Recreational		6 April–28 August[e]	Hook-and-line (14 CCR § 27.80)
Market squid (*Loligo opalescens*)	Commercial	PB<br>CP<br>SJ	1 April–31 December (14 CCR § 149)	Purse seine, drum seine, lampara nets, dip and scoop nets (14 CCR § 149.1)

Common commercial and recreational fisheries with seasonal closures at applicable sites (Piedras Blancas = PB, Campus Point = CP, South La Jolla = SJ). Active dates (season was open) used in analysis have been adjusted from published regulations where noted.

[a]Season opening date adjusted from 2 October to 1 October to account for trap setting 24 hours in advance (14 CCR § 122.2)

[b]Pursuant to Fish and Game Code Section 8276.1(c), season dates were altered from published regulations. Altered season opening date adjusted from 22 November to 21 November to account for trap setting 18 hours in advance (see citations [1] and [2] below).

[c]Season opening date adjusted from 1 August to 31 July to account for trap setting 18 hours in advance (14 CCR § 180.1)
[d]Season opening date adjusted from 31 October to 30 October to account for trap setting 18 hours in advance (14 CCR § 180.1)
[e]Open seasons defined by Pacific Fishery Management Council (see citation [3] below)

1. California Department of Fish and Wildlife, Declaration of fishery closure due to significant risk of marine life entanglement in the Dungeness crab commercial fishery, https://nrm.dfg.ca.gov/FileHandler.ashx?DocumentID=166232, 2019 (accessed 15 February 2021).
2. California Department of Fish and Wildlife, Declaration of season delay due to significant marine life entanglement risk in the Dungeness crab commercial fishery, https://nrm.dfg.ca.gov/FileHandler.ashx?DocumentID=174992, 2019 (accessed 15 February 2021).
3. Pacific Fishery Management Council, Review of 2019 Ocean Salmon Fisheries: Stock Assessment and Fishery Evaluation Document for the Pacific Coast Salmon Fishery Management Plan, Pacific Fishery Management Council, Portland, Oregon. https://www.pcouncil.org/documents/2020/02/review-of-2019-ocean-salmon-fisheries.pdf/, 2020 (accessed 23 February 2021).
