# Supplementary material for: Coastal radar as a tool for continuous and fine-scale monitoring of vessel activities of interest in the vicinity of marine protected areas
Source: PLoS One. 2022 Jul 15;17(7):e0269490. doi: 10.1371/journal.pone.0269490 (PMC9286260; doi:10.1371/journal.pone.0269490)
Supplement: S1 Appendix — (PDF) [file pone.0269490.s003.pdf]

S1 Appendix. Data preparation and false target identification details.

Day counts removed during initial data preparation at each step

	Piedras Blancas	Campus Point	South La Jolla
**Total**	365	365	365
**Offline**	113	7	35
**Maintenance**	16	26	11
**Analysis days**	236	332	319

Analysis days by month

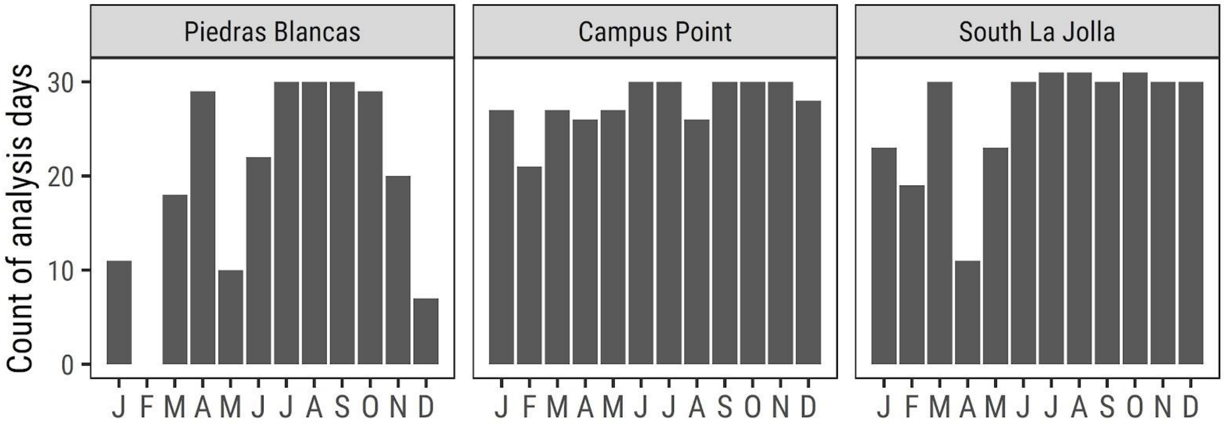

False target identification

M2 employs a model developed by machine learning that uses trajectory-based inputs to predict the likelihood that a target detected and tracked by the radar system was a true vessel and not a false target. Capitalizing on trajectory-based inputs that are not location-specific, a model is generalizable across all M2 sites. Thus, data used for model development are captured at all M2 sites. M2 users identify true vessels and false targets they observe over time using raw radar imagery, local knowledge, and target photos when available, and these data are integrated for model training.

Following the process detailed in [1] an Extreme Gradient Boosting (XGBoost) model classified false target records in M2 data. First, 4-fold cross-validation was performed on all M2 trajectory records from 1 January 2020 through 1 June 2020 identified as true vessels or false targets to train, tune, and test a model for prediction accuracy. A bootstrap method was applied where this process was repeated 10 times so that results reflect an average and standard deviation of prediction accuracy. Cross-validation results indicated 92.8 ± 0.3% accuracy (both true vessels

and false targets classified correctly). Next, the full dataset from 2020 was used to train and tune a single model and then applied to all data collected on analysis days in 2019 at the Piedras Blancas, Campus Point, and South La Jolla sites. As a result, each trajectory record was assessed a model output value ranging from 0 to 1 where values greater than 0.5 indicated trajectories likely to reflect those of true vessels.

1. Cope S, Zetterlind V, Tougher B. Integrating marine radar in a multi-sensor platform for remote, unsupervised vessel tracking in the nearshore environment. In: Global Oceans 2020: Singapore – U.S. Gulf Coast; 2020. doi: 10.1109/IEEECONF38699.2020.9389460.

Accuracy assessment

To evaluate whether prediction accuracy in the 2019 data utilized in this research was similar to the previous cross-validation results, an accuracy assessment was performed on model prediction results replicated at all three sites. The 2 days within each calendar quarter (January-March, April-June, July-September, October-December) with the highest and lowest average daily wind speed were selected for evaluation to ensure representation of different conditions. Each trajectory record from those days was evaluated as that of a true vessel or false target using qualitative analysis of trajectory shape, raw radar imagery, and target photos when available. Wind speed data were collected from the nearest data buoy station (https://www.ndbc.noaa.gov/) to a given site.

Days selected for accuracy assessment

**Study site**	**Station**	**Lat/long**	**Quarter**	**Date**	**Daily wind speed (m/s)**
**Piedras Blancas**	PSLC1	35.169 N, 120.754 W	1	19 March	1.02 ± 1.05
				30 March	6.85 ± 1.35
			2	23 April	1.01 ± 0.78
				22 May	6.02 ± 2.14
			3	14 September	0.93 ± 0.75
				21 September	4.13 ± 3.49
			4	4 November	0.75 ± 0.67
				2 October	4.41 ± 3.11
**Campus Point**	NTBC1	34.405 N, 119.692 W	1	20 January	1.30 ± 0.79
				14 January	7.66 ± 2.57
			2	10 May	1.32 ± 0.87
				17 May	5.29 ± 1.82
			3	9 September	1.50 ± 0.85
				5 August	3.77 ± 1.81
			4	3 December	1.20 ± 0.66
				1 December	4.35 ± 1.52
**South La Jolla**	LJPC1	32.867 N, 117.257 W	1	28 January	0.51 ± 0.46
				14 February	9.11 ± 4.88
			2	1 April	0.90 ± 0.72
				20 May	6.85 ± 1.23
			3	14 September	0.79 ± 0.46
				29 September	4.09 ± 1.10
			4	9 November	0.50 ± 0.35
				20 November	6.89 ± 2.02

Results of the assessment showed that overall prediction accuracy on the selected days was 94.9% (Piedras Blancas: 96.5%, Campus Point: 96.7%, South La Jolla: 92.8%). Finally, all trajectory records on analysis days in 2019 with a model output value less than 0.5 were removed from consideration as it was highly likely that these records were false targets.

Accuracy assessment results

		Predicted		
		True vessel	False target	
Actual	True vessel	598	30	95.2%
	False target	27	466	94.5%
		95.7%	94.0%	94.9%

Confusion matrix shows the number of correctly and incorrectly classified trajectories in the accuracy assessment.
