# Supplementary material for: Coastal radar as a tool for continuous and fine-scale monitoring of vessel activities of interest in the vicinity of marine protected areas
Source: PLoS One. 2022 Jul 15;17(7):e0269490. doi: 10.1371/journal.pone.0269490 (PMC9286260; doi:10.1371/journal.pone.0269490)
Supplement: S2 Appendix — (PDF) [file pone.0269490.s004.pdf]

S2 Appendix. Daily activity detailed results.

Day counts when activity of interest occurred

		Piedras Blancas	Campus Point	South La Jolla
**Focal**	Inner MPA region	93 (39.4%)	247 (74.4%)	275 (86.2%)
	Boundary MPA region	54 (22.9%)	188 (56.6%)	242 (75.9%)
	Outer MPA region	88 (37.3%)	279 (84.0%)	292 (91.5%)
**Linear**	Inner MPA region	137 (58.1%)	249 (75.0%)	282 (88.4%)
	Boundary MPA region	102 (43.2%)	219 (66.0%)	254 (79.6%)
	Outer MPA region	141 (59.7%)	295 (88.9%)	300 (94.0%)

Day counts when the total daily activity of interest (hours per km^2) was greater than zero and the corresponding percent of the total analysis days (Piedras Blancas: n = 236, Campus Point: n = 332, South La Jolla: n = 319).

Summary of daily activity normalized by area

	Piedras Blancas		Campus Point		South La Jolla	
	**Sum**	**Daily**	**Sum**	**Daily**	**Sum**	**Daily**
**All observed**	8.59	0.04 ± 0.04	34.36	0.10 ± 0.05	39.22	0.12 ± 0.07
Potential fishing	3.94	0.02 ± 0.02	12.73	0.04 ± 0.03	18.11	0.06 ± 0.04
Focal	2.05	0.01 ± 0.01	8.46	0.03 ± 0.02	11.72	0.04 ± 0.03
Linear	1.90	0.01 ± 0.01	4.27	0.01 ± 0.01	6.39	0.02 ± 0.02
**Outside MPA vicinity**	8.39	0.04 ± 0.04	29.31	0.09 ± 0.05	32.81	0.10 ± 0.07
Potential fishing	4.29	0.02 ± 0.03	10.16	0.03 ± 0.03	15.34	0.05 ± 0.04
Focal	2.24	0.01 ± 0.02	6.31	0.02 ± 0.02	9.39	0.03 ± 0.03
Linear	2.06	0.01 ± 0.01	3.85	0.01 ± 0.01	5.95	0.02 ± 0.02
**MPA vicinity**	8.82	0.04 ± 0.04	42.25	0.13 ± 0.08	49.30	0.15 ± 0.10
Potential fishing	3.49	0.02 ± 0.03	16.67	0.05 ± 0.05	22.30	0.07 ± 0.06
Focal	1.81	0.01 ± 0.02	11.86	0.04 ± 0.04	15.42	0.05 ± 0.05
Linear	1.68	0.01 ± 0.01	4.81	0.01 ± 0.02	6.87	0.02 ± 0.02
**Inner MPA region**	7.89	0.03 ± 0.03	38.74	0.12 ± 0.08	48.95	0.15 ± 0.13
Potential fishing	2.13	0.01 ± 0.02	8.62	0.03 ± 0.04	18.90	0.06 ± 0.08
Focal	0.64	0.00 ± 0.01	4.39	0.01 ± 0.04	12.88	0.04 ± 0.07
Linear	1.49	0.01 ± 0.01	4.23	0.01 ± 0.02	6.02	0.02 ± 0.02
**Boundary MPA region**	10.79	0.05 ± 0.07	46.89	0.14 ± 0.18	51.60	0.16 ± 0.17
Potential fishing	4.50	0.02 ± 0.06	21.72	0.07 ± 0.15	24.20	0.08 ± 0.13
Focal	2.93	0.01 ± 0.05	18.69	0.06 ± 0.14	19.31	0.06 ± 0.12
Linear	1.57	0.01 ± 0.02	3.03	0.01 ± 0.01	4.89	0.02 ± 0.02
**Outer MPA region**	9.66	0.04 ± 0.06	44.90	0.13 ± 0.09	48.53	0.15 ± 0.10
Potential fishing	5.10	0.02 ± 0.04	22.65	0.07 ± 0.07	23.45	0.07 ± 0.06
Focal	3.24	0.01 ± 0.03	17.50	0.05 ± 0.06	16.29	0.05 ± 0.05
Linear	1.86	0.01 ± 0.02	5.15	0.02 ± 0.02	7.16	0.02 ± 0.02

Total daily activity normalized by area (daily hours per km^2) on analysis days at each site. Average daily activity with standard deviation is also shown.

Activity of interest percent values

		Piedras Blancas	Campus Point	South La Jolla
**Focal**	Outside MPA vicinity	55.31%	34.73%	37.84%
	MPA vicinity	44.69%	65.27%	62.16%
	Inner MPA region	9.39%	10.82%	26.57%
	Boundary MPA region	43.07%	46.06%	39.83%
	Outer MPA region	47.54%	43.12%	33.60%
**Linear**	Outside MPA vicinity	54.98%	44.46%	46.42%
	MPA vicinity	45.02%	55.54%	53.58%
	Inner MPA region	30.28%	34.08%	33.33%
	Boundary MPA region	31.91%	24.41%	27.04%
	Outer MPA region	37.81%	41.50%	39.63%
