# Supplementary material for: Coastal radar as a tool for continuous and fine-scale monitoring of vessel activities of interest in the vicinity of marine protected areas
Source: PLoS One. 2022 Jul 15;17(7):e0269490. doi: 10.1371/journal.pone.0269490 (PMC9286260; doi:10.1371/journal.pone.0269490)
Supplement: S3 Appendix — (PDF) [file pone.0269490.s005.pdf]

S3 Appendix. Temporal analysis sample sizes at all sites.

Temporal analysis sample sizes at Piedras Blancas

		Inner		Boundary		Outer	
**Day/night**		74/27	102/76	46/9	73/56	74/18	112/75
**Weekday/weekend**		56/37	88/49	27/27	66/36	48/40	92/49
**Closed/open**							
Pink shrimp	Overall		24/113		17/85		24/117
	Day		15/87		9/64		14/98
	Night		16/60		10/46		16/59
	Weekday		14/74		11/55		15/77
	Weekend		10/39		6/30		9/40
Dungeness crab	Overall	73/20		48/6		75/13	
	Day	58/16		41/5		65/9	
	Night	22/5		8/1*		14/4	
	Weekday	46/10		25/2		41/7	
	Weekend	27/10		23/4		34/6	
Dungeness crab (rec)	Overall	38/55		24/30		36/52	
	Day	30/44		22/24		30/44	
	Night	11/16		2/7		7/11	
	Weekday	24/32		12/15		20/28	
	Weekend	14/23		12/15		16/24	
Spot prawn	Overall	23/70		16/38		27/61	
	Day	19/55		12/34		25/49	
	Night	7/20		5/4		5/13	
	Weekday	15/41		10/17		15/33	
	Weekend	8/29		6/21		12/28	
Nearshore fishery	Overall	17/76	21/116	8/46	12/90	16/72	18/123
	Day	14/60	19/83	7/39	8/65	12/62	15/97
	Night	3/24	6/70	1/8	5/51	4/14	9/66
	Weekday	9/47	13/75	3/24	7/59	9/39	12/80
	Weekend	8/29	8/41	5/22	5/31	7/33	6/43
Groundfish (rec)	Overall	6/87	8/129	1/53	5/97	5/83	8/133

	Day	4/70	6/96	1/45	3/70	2/72	4/108
	Night	2/25	4/72	0/9*	2/54	3/15	7/68
	Weekday	3/53	5/83	1/26	3/63	3/45	5/87
	Weekend	3/34	3/46	0/27*	2/34	2/38	3/46
Ridgeback prawn	Overall		69/68		56/46		75/66
	Day		53/49		44/29		62/50
	Night		38/38		28/28		37/38
	Weekday		42/46		35/31		46/46
	Weekend		27/22		21/15		29/20
Salmon	Overall	73/20	108/29	41/13	80/22	66/22	110/31
	Day	58/16	78/24	36/10	54/19	53/21	85/27
	Night	20/7	62/14	5/4	47/9	14/4	60/15
	Weekday	42/14	69/19	18/9	52/14	34/14	72/20
	Weekend	31/6	39/10	23/4	28/8	32/8	38/11
Salmon (rec)	Overall	93/43	137/66	54/30	102/49	88/47	141/65
	Day	74/37	102/55	46/25	73/39	74/43	112/56
	Night	27/9	76/31	9/6	56/23	18/7	75/31
	Weekday	56/25	88/40	27/16	66/30	48/26	92/41
	Weekend	37/18	49/26	27/14	36/19	40/21	49/24
Market squid	Overall	6/87		1/53		5/83	
	Day	4/70		1/45		2/72	
	Night	2/25		0/9*		3/15	
	Weekday	3/53		1/26		3/45	
	Weekend	3/34		0/27*		2/38	

Focal activity analysis shown in white; linear activity analysis shown in grey shading. Fishing gear/method not applicable where blank. Asterisks (*) indicate small sample sizes that prevented temporal analysis.

Temporal analysis sample sizes at Campus Point

		Inner		Boundary		Outer	
**Day/night**		224/86	223/132	176/29	201/82	271/104	285/132
**Weekday/weekend**		174/73	178/71	139/49	155/64	198/81	211/84
**Closed/open**							
Spiny lobster	Overall	142/105		91/97		148/131	
	Day	131/93		84/92		146/125	
	Night	39/47		12/17		49/55	
	Weekday	100/74		67/72		102/96	
	Weekend	42/31		24/25		46/35	
Spiny lobster (rec)	Overall	139/108		90/98		146/133	
	Day	128/96		83/93		144/127	
	Night	38/48		12/17		49/55	
	Weekday	99/75		67/72		101/97	
	Weekend	40/33		23/26		45/36	
Spot prawn	Overall	63/184		55/133		73/206	
	Day	54/170		52/124		69/202	
	Night	32/54		12/17		34/70	
	Weekday	46/128		43/96		53/145	
	Weekend	17/56		12/37		20/61	
Nearshore fishery	Overall	26/221	29/220	22/166	27/192	45/234	45/250
	Day	25/199	28/195	21/155	23/178	45/226	44/241
	Night	2/84	8/124	2/27	7/75	13/91	12/120
	Weekday	16/158	18/160	14/125	16/139	31/167	30/181
	Weekend	10/63	11/60	8/41	11/53	14/67	15/69
Groundfish (rec)	Overall	27/220	19/230	33/155	24/195	37/242	39/256
	Day	25/199	15/208	33/143	21/180	36/235	37/248
	Night	3/83	5/127	2/27	6/76	7/97	14/118
	Weekday	20/154	15/163	25/114	18/137	28/170	29/182
	Weekend	7/66	4/67	8/41	6/58	9/72	10/74
Ridgeback prawn	Overall		104/145		86/133		105/190
	Day		95/128		82/119		104/181

	Night		53/79		31/51		39/93
	Weekday		73/105		61/94		75/136
	Weekend		31/40		25/39		30/54
White seabass	Overall	56/191	58/191	36/152	48/171	72/207	74/221
	Day	49/175	54/169	34/142	44/157	72/199	73/212
	Night	12/74	20/112	5/24	10/72	23/81	24/108
	Weekday	38/136	38/140	24/115	30/125	49/149	50/161
	Weekend	18/55	20/51	12/37	18/46	23/58	24/60
California halibut	Overall		58/191		48/171		74/221
	Day		54/169		44/157		73/212
	Night		20/112		10/72		24/108
	Weekday		38/140		30/125		50/161
	Weekend		20/51		18/46		24/60
Market squid	Overall	40/207		46/142		61/218	
	Day	38/186		46/130		60/211	
	Night	4/82		3/26		15/89	
	Weekday	27/147		32/107		44/154	
	Weekend	13/60		14/35		17/64	

Focal activity analysis shown in white; linear activity analysis shown in grey shading. Fishing gear/method not applicable where blank.

Temporal analysis sample sizes at South La Jolla

		Inner		Boundary		Outer	
**Day/night**		269/93	278/115	236/59	252/74	288/126	297/155
**Weekday/weekend**		191/84	198/84	168/74	177/77	207/85	214/86
**Closed/open**							
Spiny lobster	Overall	146/129		122/120		159/133	
	Day	144/125		121/115		158/130	
	Night	40/53		18/41		55/71	
	Weekday	101/90		85/83		111/96	
	Weekend	45/39		37/37		48/37	
Spiny lobster (rec)	Overall	143/132		122/120		155/137	
	Day	142/127		121/115		154/134	
	Night	37/56		18/41		55/71	
	Weekday	100/91		85/83		109/98	
	Weekend	43/41		37/37		46/39	
Spot prawn	Overall	69/206		65/177		71/221	
	Day	66/203		62/174		69/219	
	Night	31/62		24/35		40/86	
	Weekday	48/143		45/123		50/157	
	Weekend	21/63		20/54		21/64	
Nearshore fishery	Overall	22/253	21/261	15/227	13/241	32/260	34/266
	Day	20/249	19/259	15/221	12/240	32/256	34/263
	Night	7/86	10/105	2/57	3/71	5/121	6/149
	Weekday	13/178	12/186	9/159	7/170	22/185	23/191
	Weekend	9/75	9/75	6/68	6/71	10/75	11/75
Groundfish (rec)	Overall	35/240	38/244	33/209	34/220	39/253	40/260
	Day	34/235	38/240	31/205	34/218	37/251	39/258
	Night	9/84	14/101	7/52	10/64	17/109	19/136
	Weekday	27/164	30/168	25/143	26/151	31/176	32/182
	Weekend	8/76	8/76	8/66	8/69	8/77	8/78
Ridgeback prawn	Overall		118/164		105/149		121/179
	Day		117/161		105/147		120/177

	Night		39/76		28/46		62/93
	Weekday		83/115		73/104		85/129
	Weekend		35/49		32/45		36/50
White seabass	Overall	48/227	48/234	41/201	41/213	58/234	62/238
	Day	47/222	47/231	41/195	41/211	58/230	62/235
	Night	13/80	16/99	4/35	7/67	18/108	17/138
	Weekday	32/159	31/167	27/141	27/150	39/168	42/172
	Weekend	16/68	17/67	14/60	14/63	19/66	20/66
Market squid	Overall	52/223		45/197		62/230	
	Day	49/220		42/193		60/228	
	Night	15/78		9/50		20/106	
	Weekday	37/154		32/136		46/161	
	Weekend	15/69		13/61		16/69	

Focal activity analysis shown in white; linear activity analysis shown in grey shading. Fishing gear/method not applicable where blank.
